# Supplementary material for: Advanced methods for gene network identification and noise decomposition from single-cell data
Source: Nat Commun. 2024 Jun 8;15:4911. doi: 10.1038/s41467-024-49177-1 (PMC11162465; doi:10.1038/s41467-024-49177-1)
Supplement: Supplementary file 3 — Reporting Summary [file 41467_2024_49177_MOESM3_ESM.pdf]

nature portfolio

Corresponding author(s): Mustafa Khammash

Last updated by author(s): Nov 2, 2023

Reporting Summary

Nature Portfolio wishes to improve the reproducibility of the work that we publish. This form provides structure for consistency and transparency in reporting. For further information on Nature Portfolio policies, see our Editorial Policies and the Editorial Policy Checklist.

Statistics

For all statistical analyses, confirm that the following items are present in the figure legend, table legend, main text, or Methods section.

n/a	Confirmed	
☐	☒	The exact sample size (*n*) for each experimental group/condition, given as a discrete number and unit of measurement
☐	☒	A statement on whether measurements were taken from distinct samples or whether the same sample was measured repeatedly
☒	☐	The statistical test(s) used AND whether they are one- or two-sided<br>*Only common tests should be described solely by name; describe more complex techniques in the Methods section.*
☒	☐	A description of all covariates tested
☒	☐	A description of any assumptions or corrections, such as tests of normality and adjustment for multiple comparisons
☐	☒	A full description of the statistical parameters including central tendency (e.g. means) or other basic estimates (e.g. regression coefficient) AND variation (e.g. standard deviation) or associated estimates of uncertainty (e.g. confidence intervals)
☒	☐	For null hypothesis testing, the test statistic (e.g. *F*, *t*, *r*) with confidence intervals, effect sizes, degrees of freedom and *P* value noted<br>*Give P values as exact values whenever suitable.*
☐	☒	For Bayesian analysis, information on the choice of priors and Markov chain Monte Carlo settings
☐	☒	For hierarchical and complex designs, identification of the appropriate level for tests and full reporting of outcomes
☒	☐	Estimates of effect sizes (e.g. Cohen's *d*, Pearson's *r*), indicating how they were calculated

Our web collection on statistics for biologists contains articles on many of the points above.

Software and code

Policy information about availability of computer code

Data collection	For experimental data, we use the protocol in (Rullan et al., Molecular Cell, 2018) and (Kumar et al., Nature Communications, 2021)<br>1. YouScope 2.0 (build: R2016-01, open source) -- Used for microscope and projector control.<br>2. MATLAB R2016a (academic use) -- Used for experiment run.
Data analysis	All analyses in this study were performed on MATLAB R2019a (academic use) and Python using custom scripts. Manuscript figures were structured and formatted on Adobe Illustrator.<br><br>The custom code is available at:<br>https://github.com/ZhouFang92/Rao-Blackwellized-CME-solver<br>https://github.com/ZhouFang92/Rao-Blackwell-method-for-cell-specific-model-identification

For manuscripts utilizing custom algorithms or software that are central to the research but not yet described in published literature, software must be made available to editors and reviewers. We strongly encourage code deposition in a community repository (e.g. GitHub). See the Nature Portfolio guidelines for submitting code & software for further information.

Data

Policy information about availability of data

All manuscripts must include a data availability statement. This statement should provide the following information, where applicable:

- Accession codes, unique identifiers, or web links for publicly available datasets
- A description of any restrictions on data availability
- For clinical datasets or third party data, please ensure that the statement adheres to our policy

The data for all the results/figures presented in this article are available in
1. https://github.com/ZhouFang92/Rao-Blackwell-method-for-cell-specific-model-identification/tree/main/Example_of_model_identification/results_in_paper
2. https://github.com/ZhouFang92/Rao-Blackwell-method-for-cell-specific-model-identification/tree/main/Example_of_model_identification/real_data

Research involving human participants, their data, or biological material

Policy information about studies with human participants or human data. See also policy information about sex, gender (identity/presentation), and sexual orientation and race, ethnicity and racism.

Reporting on sex and gender	Not applicable. We didn't use human cells; only yeast cells are investigated.
Reporting on race, ethnicity, or other socially relevant groupings	Not applicable. Only yeast cells are investigated.
Population characteristics	Not applicable. Only yeast cells are investigated.
Recruitment	Not applicable. Only yeast cells are investigated.
Ethics oversight	Not applicable. Only yeast cells are investigated.

Note that full information on the approval of the study protocol must also be provided in the manuscript.

Field-specific reporting

Please select the one below that is the best fit for your research. If you are not sure, read the appropriate sections before making your selection.

[x] Life sciences   [ ] Behavioural & social sciences   [ ] Ecological, evolutionary & environmental sciences

For a reference copy of the document with all sections, see nature.com/documents/nr-reporting-summary-flat.pdf

Life sciences study design

All studies must disclose on these points even when the disclosure is negative.

Sample size	We investigated all the 130 yeast cells whose RNA dynamics are recorded under the microscope over a time horizon of 4 hours.
Data exclusions	No data were excluded.
Replication	Our method was successful in identifying all these 130 cells.
Randomization	Not applicable. We didn't allocate the cells into groups. Our method was applied to each cell.
Blinding	Not applicable. Our research didn't allocate the cells into groups. Our method was applied to each cell.

Reporting for specific materials, systems and methods

We require information from authors about some types of materials, experimental systems and methods used in many studies. Here, indicate whether each material, system or method listed is relevant to your study. If you are not sure if a list item applies to your research, read the appropriate section before selecting a response.

Materials & experimental systems

n/a	Involved in the study
☒	☐ Antibodies
☒	☐ Eukaryotic cell lines
☒	☐ Palaeontology and archaeology
☒	☐ Animals and other organisms
☒	☐ Clinical data
☒	☐ Dual use research of concern
☒	☐ Plants

Methods

n/a	Involved in the study
☒	☐ ChIP-seq
☒	☐ Flow cytometry
☒	☐ MRI-based neuroimaging

Plants

Seed stocks	Not applicable.
Novel plant genotypes	Not applicable.
Authentication	Not applicable.
